# Physic's Lenten Pastoral

**Published:** 1893-02-25

**Authors:** 


### The Hospital

An Institutional Journal of

**Science, Medicine, Nursing, and Philanthropy.**

*For Hospitals, Asylums, Infirmaries, Dispensaries, Medical Practitioners, Students, Nurses, and the Charitable Public.*

Vol. XIII.—No. 335.] [Feb. 25, 1893.

### Physic's Lenten Pastoral

The physician is like the clergyman, he has a great deal to do with persons. His experience of men, women, and children makes him patient, tolerant, and even humble. Seeing in others so much that is weak, so much that is pitiful, he gradually begins to think that others may sometimes see in him weaknesses and pitifulnesses. This is a hopeful frame of mind, and very scientific: it leads down to the roots of things, and not only to the roots, but outwards to the remotest branches. On one metaphysical question the physician gradually becomes a very convinced believer—he believes in the "complexity" of human nature. The ancients divided man into body, soul, and spirit. The experienced modern physician sometimes feels that he cannot pack all the phenomena of human nature into anything like so limited a number of elemental characteristics: he feels as if he must have six or seven elements at least, instead of merely three.

It is personality that makes human life interesting. Compared with this, matter, mechanism, and even chemical and vital forces, are nowhere. It is the person, the mind, the power of mental emotion, that makes age youthful and youth aged. The physician who takes a mechanically scientific view of human nature is entirely out of the running. He shirks the real problem he is called upon to solve. Chemical action takes us a certain distance, and "vital force," if there be such a thing in ultimate analysis, may explain much. But the true physician recognises the elements of mind, of soul, of emotion, of personality, of individuality. Without such recognition the cleverest must be a dull and stupid bungler.

Last Sunday was the first Sunday in Lent. What has physic to do with Lent? Much: everything! Lent, when properly used, is an admirable six weeks' tonic both for body and mind. "It is the sorriest of intellects," said a well-known clergyman last Sunday, "that does not sometimes pause, and, taking its stand in the midst of things, ask itself—Why, Whence, Whither?" A bull or an elephant probably never brings himself to such a pause. But a mind—a Shakespeare, a Darwin, a Huxley, a Tennyson—must have asked himself such a question a million times over. The pause, the mental effort, is bracing to the mind; and whatever braces the mind braces the body in at least an equal degree. The most efficient of all tonics for the body is a firm and vigorous mind.

The intelligent physician thinks well of Lent, then. If he be an Englishman he loves to turn it to practical account. This is in accordance with his nature; in accordance with the part assigned to him in the present ordering of the world. The great example which the Englishman—the Briton rather—has shown to the world for many centuries, and is still showing, is the example of a sublime practicality; not a dull and mechanical practicality, but an intellectual and highly rational practicality. The Englishman strives to master every theory in the Universe, not for the mere sake of the theory, but in order that he may chain it to the car of practice and noble deeds.

What is the real, actual, and innermost significance of Lent? An Oriental might answer this question by prostrating himself upon the ground in an attitude of profound self-abasement, and covering his head with ashes. That does not commend itself to the English mind, except in a limited number of cases. The Briton looks deeper. The striking of an attitude may mean much, or it may mean nothing at all. What does Lent mean to us? It means something equivalent to a forty days' stern conflict in the wilderness. It means a choice between the soul and the body. Is mind or is matter the paramount thing? Is intellect or flesh the chief characteristic of man? Is man a mind, a conscience, a will, a personality; or is he a fleshly body of instincts obeying an environment? It is all there.

It is persons who take themselves so seriously as this that the British physician has to deal with every day of his life. Now it is a man, now a woman; sometimes a young man whose world is peopled with the grossest and vilest of devils; sometimes a young woman, whose subtler temptations insidiously undermine and impair the energy of both body and mind. What is to be done, for example, when a mother, whose own mind and character have been formed and steadied by years of stern duty in her home and family, brings her daughter, all limp in body and mind, from the reading of the literary trivialities of the time, or from the daily routine of stupid frivolities more mindless and imbecile still, into the physician's consulting room? It is not a simple task the physician is called upon to perform here. Unconsciously he reverts to first principles; he sees that his patient has no mind, no character, no conscience; there is nothing to work upon. Here is one cf the proofs that it is mind which lies at the root of all things. How shall the physician set to work to cure this youthful wreck? What would he do if he could? Would he not treat mind as well as body? Would he not prescribe a course of careful mental discipline? Would he not order a wholesome and self-denying life, as well as his potions, his powders, and his pills? Most assuredly he would. Mend this young woman's mind, and her body, in nine cases out of ten, will promptly mend itself. Let her become the intelligent helper of the needy; let her soothe the distresses of others and forget her own. Let this be done intelligently, systematically, daily, and with resolute purpose, and in a very few weeks the cure of the patient's ills will be complete.

This regimen is the fitting cure for young men as well as for young women when the causes of their ills are similar—idleness and frivolity. Fortunately, young men, for the most part, have to work, and those who work at least escape the ills of an imbecile *ennui*, or a worse than imbecile triviality of occupation. But there are a great many men and women in this country of a different class. These, though past middle life, have still a good many years to live, and their ample means render them a prey to all the ills of a withering and degrading idleness. Such persons as these crowd the consulting-rooms of prominent physicians. Life has become a burden to them; and yet they would give worlds to preserve it. Nothing terrifies them so much as illness; and yet they have not the resolution which, if used, would drive all illness away. In such cases the physician often orders physic when he would fain prescribe faith, and drugs when he knows that duty would be the real means of cure. To such we venture to prescribe a Lenten regimen.

Self-denial is the note of Lent; and it is the note of all high life. It needs no logic to show that self-denial which helps the distressed is of much more value than self-denial which begins and ends with self. The true Englishman does not value self-denial which ends in nothing; he does value that which leads to a solid, practical result. But even the Englishman is not always practical enough to set himself upon useful forms of self-denial. It is our province to know of such useful forms of self-denial, and our privilege to point out the way. Needless to say we stand for hospitals; and we urge with all possible earnestness the claims of a "Hospital Lent." During the forty days' temptation, the Founder of Christianity put aside the glamour of "all the kingdoms of the world and the glory of them," in order that for the rest of his life he might serve the poor. If any readers of similar mood elect to serve the poor by making this a "Hospital Lent," but are doubtful about the methods, we shall be glad to hear from them, publicly or privately, and to show them the way.

